# Host APOBEC3G Protein Inhibits HCV Replication through Direct Binding at NS3

**DOI:** 10.1371/journal.pone.0121608

**Published:** 2015-03-26

**Authors:** Yan-Ping Zhu, Zong-Gen Peng, Zhou-Yi Wu, Jian-Rui Li, Meng-Hao Huang, Shu-Yi Si, Jian-Dong Jiang

**Affiliations:** 1 Laboratory of Antiviral Research, Institute of Medicinal Biotechnology, Chinese Academy of Medical Sciences/Peking Union Medical College, Beijing, 100050, China; 2 State Key Laboratory of Bioactive Substance and Function of Natural Medicines, Institute of Materia Medica, Chinese Academy of Medical Sciences/Peking Union Medical College, Beijing, 100050, China; University of Modena & Reggio Emilia, ITALY

## Abstract

Human APOBEC3G (hA3G) is a cytidine deaminase that restricts replication of certain viruses. We have previously reported that hA3G was a host restriction factor against hepatitis C virus (HCV) replication, and hA3G stabilizers showed a significant inhibitory activity against HCV. However, the molecular mechanism of hA3G against HCV remains unknown. We show in this study that hA3G’s C-terminal directly binds HCV non-structural protein NS3 at its C-terminus, which is responsible for NS3’s helicase and NTPase activity. Binding of hA3G to the C-terminus of NS3 reduced helicase activity, and therefore inhibited HCV replication. The anti-HCV mechanism of hA3G appeared to be independent of its deamination activity. Although early stage HCV infection resulted in an increase in host hA3G as an intracellular response against HCV replication, hA3G was gradually diminished after a long-term incubation, suggesting an unknown mechanism(s) that protects HCV NS3 from inactivation by hA3G. The process represents, at least partially, a cellular defensive mechanism against HCV and the action is mediated through a direct interaction between host hA3G and HCV NS3. We believe that understanding of the antiviral mechanism of hA3G against HCV might open an interesting avenue to explore hA3G stabilizers as a new class of anti-HCV agents.

## Introduction

Human cellular protein apolipoprotein B messenger RNA (mRNA)-editing enzyme catalytic polypeptide-like 3G (human APOBEC3G, or hA3G) is a cytidine deaminase that interrupts the life cycle of certain viruses [1, 2]. The defensive function of hA3G was discovered first in cells infected with human immunodeficiency virus type 1 (HIV-1) [3], and the antiviral mechanism of hA3G is mainly associated with induction of G/A hypermutation in HIV-1 genome [4, 5]. On the other hand, HIV-1 Vif binds hA3G in the cytoplasm, forming the Vif-Cul5-SCF complex which facilitates ubiquitination and subsequent degradation of hA3G by proteosomes [6]. Our previous report showed that hA3G is also a cellular restriction factor against hepatitis C virus (HCV) replication [7]. Treatment of HCV-infected Huh7.5 cells with RN-5 or IMB-26 (known hA3G stabilizing compounds [8, 9]) increased intracellular hA3G and accordingly inhibited HCV replication. The compounds inhibited HCV replication through increasing the level of hA3G incorporated into HCV viral particles [7]. Interestingly, G/A hypermutation in HCV genome was not detected in the cells treated with the compounds [7], suggesting existence of an unknown antiviral mechanism of hA3G. The phenomenon appears to be consistent with reports from other groups studying hepatitis B virus, adeno-associated virus, measles, mumps and respiratory syncytial virus [10–12] and it does pique strong curiosity. In searching for the antiviral mechanism of hA3G, the interaction between hA3G and HCV was investigated, through which we found that cellular hA3G directly bound HCV non-structural protein NS3, through which the activity of NS3’s helicase was decreased. What described below are the results demonstrating the direct interaction between host hA3G enzyme and HCV NS3 protein.

## Materials and Methods

### Plasmids

The plasmids phA3G-HA, phA3G-HA (aa 157–384) and phA3G-HA (aa 1–156) were generously provided by Dr. Shan Cen at the Lady Davis Institute for Medical Research and McGill University AIDS Centre, which expressed full length wild-type hA3G, hA3G amino acid 157–384 and hA3G amino acid 1–156 fused with an HA tag at the C-terminus, respectively. The plasmids pNS3-His and pNS3/4A-His expressed the full-length form of HCV NS3 or NS3/4A with His tag at the C-terminus and were constructed by inserting the study sequences into a pcDNA3.1(+) vector. All above plasmids were constructed under a T7 promoter. The plasmid pFL-J6/JFH/JC1 containing the full-length chimeric HCV cDNA was kindly provided by Vertex Pharmaceuticals Inc (Boston, MA).

### Cell culture and viral infection

Huh7.5 cells (kindly provided by Vertex Pharmaceuticals Inc, Boston, MA) and 293T/17 cells (purchased from ATCC) were cultured in Dulbecco's Modified Eagle Medium (DMEM, Invitrogen, CA) supplemented with 10% inactivated fetal bovine serum (Invitrogen) and 1% penicillin-streptomycin (Invitrogen). HCV virus stock was prepared and used to infect native Huh7.5 cells at an infective dose of 45 IU/cell as described previously [13].

### Immunoprecipitation assay

HCV-infected Huh7.5 cells were collected from tissue culture flasks 48hrs after transfection with either phA3G-HA plasmid or control plasmid (pcDNA 3.1/V5/A) using FuGENE Transfection Reagents (Roche Applied Science), and were then lysed in CytoBuster Protein Extraction Reagent (Novagen) with 1mM protease inhibitor cocktail (Roche Applied Science). After centrifugation, supernatants were incubated with 2 μg of HA-specific antibody (F-7, Santa Cruz) for 16 hrs at 4°C, followed by addition of 50 μL Protein A Agarose (Roche Applied Science) and incubated for 3 hrs. The immunoprecipitates were then washed 4 times with DPBS by brief centrifugation. The pellets were resuspended with 50 μL 2× loading buffer, and then boiled for 5 min. After brief centrifugation, the resultant supernatants were analyzed with SDS-PAGE, the proteins in the gel were extracted and then sequenced by protein sequencing as following, or were detected with western blot to validate the proteins.

### Protein sequencing

The detailed method was described previously with modifications [14]. The proteins from SDS-PAGE gels were separated and performed on a Waters NanoAcquity LC with a binary buffer system. The samples were loaded onto the trap column and then separated by an initial linear gradient of buffer B. The MS was operated in Data-Dependent MS/MS Scan mode, with one full scan acquisition in the Orbitrap with an Automatic Gain Control (AGC) target value of 1× 10^6^ ions. The 20 most intensive ions with a charge >1 were selected for MS/MS analysis. CID (collision-induced dissociation) scans were collected at an AGC target value of 5,000 with a maximum injection time of 25s, and dynamic exclusion was set to 30s. The MS/MS spectra were searched on Sorcerer-SEQUEST (version 4.0.4 build, Sage-N Research, Inc.) against a composite target/decoy database. Searching parameters consisted of semitryptic restriction, fixed modification of Cys (+57.0215 Da, alkylation by iodoacetamide) and dynamic modification of oxidized Met (+15.9949 Da). Mass tolerance was set to ±20 ppm. Peptide matches were filtered by a minimal peptide length of 6 amino acids.

### Preparation of maltose-binding protein (MBP)-NS3/NS4A fusion protein and affinity chromatography assay

The maltose-binding protein (MBP)-NS3/4A fusion protein was made as described previously [15]. HCV NS3/4A fusion protein was applied onto an amylose resin column and then rinsed with washing buffer. Then the lysed supernatants prepared with CytoBuster Protein Extraction Reagent (Novagen) with 1mM protease inhibitor cocktail (Roche Applied Science) from Huh7.5 cells transfected with plasmids were applied onto the above handled column and rinsed again with washing buffer. After elution with eluting buffer, the elutriants were concentrated via Vacuum Centrifugal Concentrator (Matin), and the sample was analyzed by western blot.

### Western blot

Proteins extracted from cells or concentrated from elutriants were analyzed with SDS-PAGE, the proteins on the membrane were probed with anti-HCV Core (C7-50, Abcam Ltd.), anti-NS3 (H23, Abcam Ltd.), anti-hA3G (ab75560, Abcam Ltd.), anti-HA (6E2, Cell Signaling Biotechnology Inc.), or anti-HA-tag [HRP] (A00169, GenScript.) antibody, respectively, with anti-Actin antibody (TA-09, ZSGB-BIO, China) served as the control. After washing with TBST, the membrane was incubated with goat anti-mouse (sc-2005, Santa Cruz Biotechnology Inc.) or goat anti-rabbit (sc-2004, Santa Cruz Biotechnology Inc.) secondary antibody, respectively. Protein signals were visualized and captured using Immobilon Western Chemiluminescent HRP Substrate ECL working solution (Millipore Inc.) with ChemiDo XRS gel imager system (Bio-Rad, CA).

### Immunofluorescent staining

HCV-infected Huh7.5 cells were seeded into confocal culture dishes (NEST Biotechnology Co. Ltd.). After incubating for 24 hrs, cells were washed 3 times with ice-cold PBS, and then fixed with paraformaldehyde for 10 min and permeabilized with PBS containing 0.5% Triton X-100 for 10 min. Cells were next blocked with TBST containing 5% BSA, followed by an overnight incubation with anti-hA3G (H-63, sc-48820) and anti-HCV NS3 (H23, ab13830) or anti-HCV core (C7-50, ab2740) antibodies at 4°C. After washing 3 times with TBST, cells were probed with goat anti-rabbit Cy3 (A0516) and goat anti-mouse Alexa Fluor 488 (A0428) at room temperature for 1 hr. Then, the slide was washed 3 times with cold TBST. Cell nuclei were counterstained with DAPI for 5min at room temperature. The slide was mounted with anti-fade mounting medium and visualized using a PerkinElmer UltraVIEW VoX laser scanning spectral confocal microscope. Merged images were obtained using Volocity Demo software.

### HCV NS3 serine protease assay *in vitro*

NS3 serine protease activity was quantified by fluorescent resonance energy transfer assay [16]. The assay was performed using the Ac-Asp-Glu-Asp (EDANS)-Glu-Glu-Abu- ψ-[COO]-Ala-Ser-Lys (DABCYL)-NH_2_ (FRET-S) fluorescent peptide (AnaSpec, USA) as a substrate. Briefly, 140μL buffer A, 20μLof sample dissolved in buffer A with different concentrations and 20μL MBP-NS3/4A protease (purified as above description) diluted in buffer A were added to a 96-well plate respectively and mixed well. The reaction was initiated by adding 20μL of FRET-S, and then monitored at 37°C using an EnSpire Multimode Plate Readers (PerkinElmer Inc.) with excitation and emission filters of 355 nm and 520 nm. The inhibitory rate was calculated and normalized to the positive and negative controls.

### *In vitro* assay for HCV NS3 helicase and NTPase

Fluorescent assay was performed to measure the NS3 helicase activity [17]. The substrate [hairpin FAM top strand 5’-FAM-CCTACGCCACCAGCTCCGTAGG-BHQ-3’ and hairpin FAM bottom strand 5’-CCTACGGAGCTGGTGGCGTAGG(T)20-3’] for the assay was prepared. Standard helicase assays were performed by adding 140μL reaction buffer, 20μL of 200μM substrate and 20μL of 20 mM ATP into a 96-well plate. The unwinding reaction was started after addition of 20μL of 100μg/mL MBP-NS3/4A or NS3 protein (provided by Dr. Pei-Zhen Tao in Institute of Medicinal Biotechnology). The fluorescent signals were continuously monitored at 20°C using an EnSpire Multimode Plate Readers (PerkinElmer Inc.), with excitation and emission filters of 492 nm and 520 nm respectively. The inhibitory rate was calculated and normalized to positive and negative controls. NTPase assay was done with a colorimetric method performed in a 96-well plate using a malachite green reagent [18]. Free phosphate released from the NTPase reaction was quantified using a malachite green phosphate assay kit (BioAssay System) and performed according to the recommended procedures of manufacturer.

### BIAcore

The surface plasmon resonance experiment was performed on a BIAcore biosensor system (BIAcore T100). In brief, the carboxymethylated surface of the series S sensor chip CM5 (GE Healthcare Bio-Sciences, Sweden) was firstly activated with a mixture of N-hydroxysuccinimide (NHS) and 1-ethyl-3-(3- dimethylaminopropyl) carbodiimide hydro- chloride (EDC) (BIAcore AB). Subsequently, purified hA3G-HA was injected into flow cell 2 (FC2) for immobilization on the sensor surface. To analyze the binding of NS3-His with hA3G-HA, 160 μL of NS3-His [0.3, 0.6, 1.2 or 3 μM in HBS-EP+ buffer (GE Healthcare Bio-Sciences, Sweden)] were injected, followed by flowing the buffer over the chip and a regeneration step with Glycine-HCl (pH 1.5). Differences in resonance spectra (FC2–FC1) were recorded. Data were evaluated using the software Biacore T100 Evaluation (Biacore AB). The HCV NS3-His and hA3G-HA were purified with HisTrap HP (GE Healthcare Bio-Sciences AB, Sweden) and Pierce Anti-HA Agarose (Thermo Scientific, USA) respectively, and desalinated with HisTrap HP Desalting (GE Healthcare Bio-Sciences AB, Sweden) and Zeba Spin Desalting Columns (Thermo scientific, USA) respectively, according to the manufacturer’s operating manuals. The concentration of purified proteins were quantified with Pierce BCA Protein Assay Kit (Thermo Scientific,USA).

### Real time qRT-PCR

RNA extracted from cells with RNeasy Mini Kit (QIAGEN) was analyzed using AgPath-ID One-Step RT-PCR Kit (Applied Biosystems). Fluorescent signals were detected with 7500-fast real time PCR system (Applied Biosystems). Primer pairs of 5’-CGGGAGAGCCATAGTGGTCTGCG-3’ and 5’-CTCGCAAGCACCCTATCAGGCAG TA-3’, and TaqMan probe 5’-FAM-AGGCCTTGTGGTACTGCCT-TAMRA-3’ were for HCV; primer pairs of 5’-ggtcagaggacggcatgaga-3’ and 5’-GCAGGACCCAGG TGTCATTG-3’, and TaqMan probe 5’-FAM-CTGTGTTATGAGGTGGAGCGCA- TAMRA -3’ were for hA3G; and the primer pairs of 5’-CGGAGTCAACGGATTTGGTCG TAT-3’ and 5’-AGCCTTCTCCATGGTGGTGAAGAC-3’, and TaqMan probe 5’-FAM-CCGTCAAGG CTGAGAACGG-TAMRA-3’ were for the internal control gene, glyceraldehyde 3-phosphate dehydrogenase (GAPDH). The qRT-PCR was done according to vender’s recommendations. The results were calculated with 2^ΔΔCT^.

### Homology modeling and protein docking of hA3G to NS3

All of the computational simulations were carried out using Discovery studio 3.0 (Accelrys, San Diego, CA). A BLAST search of the amino acid sequences of HCV NS3 and hA3G were conducted against Protein Data Bank (PDB; http://www.rcsb.org) to obtain suitable templates for study of the viral strain (FL-J6/JFH/JC1) and hA3G. As the whole-sequence crystal structure of hA3G is not available yet and all structures are from those containing the C-terminus only, we selected one of the structures published in Nature as a model [19]. The homology modeling of NS3 was then performed with the crystal structure of NS3 (PDB code: 3O8B). First, we used the ZDock program to dock hA3G into NS3. Over 2000 poses were generated by ZDock and then grouped into 100 clusters. Then, three poses selected from 3 typical clusters with the highest docking scores were chosen for further refinement carried out by the RDock program. Finally, although a ZDock score of Pose1 is highest among all of the poses, we chose Pose11 as the most reliable pose for analysis because of overall consideration. For instance, in respect to the other top poses, Pose 11 owns the lowest RDock energy after refinement, a better ZRank score, as well as an improved cluster size. Thus, we considered Pose 11 as the best pose to mimic the interaction between hA3G and NS3, which was finally chosen as the suitable hA3G-NS3 complex for further study.

### RNAi experiments

Huh7.5 cells were planted into the 6-well plates and infected with HCV viral stock. After 48 hours incubation, hA3G siRNA (Santa Cruz, sc-60091) was transfected into the HCV infected cells using Lipofectamine RNAiMAX (Invitrogen), with a non-relevant control siRNA-A (Santa Cruz, sc-37007) as reference. Cells were harvested for proteins extraction 48 hours after transfection. The samples were then analyzed via western blot.

### Statistical analysis

The protein intensity of western bolt bands was scanned with Gelpro32 soft, and normalized as 1.00 for the control group. Data shown in the histogram were expressed as mean ± standard deviation of 3 independent experiments. All of the data were analyzed using a one way analysis of variance (ANOVA), and significant effect was subsequently analyzed using *student t*-test. The level of significance was set at *P*<0.05.

## Results

### Protein-protein interaction between hA3G and HCV NS3

**Fig 1:**
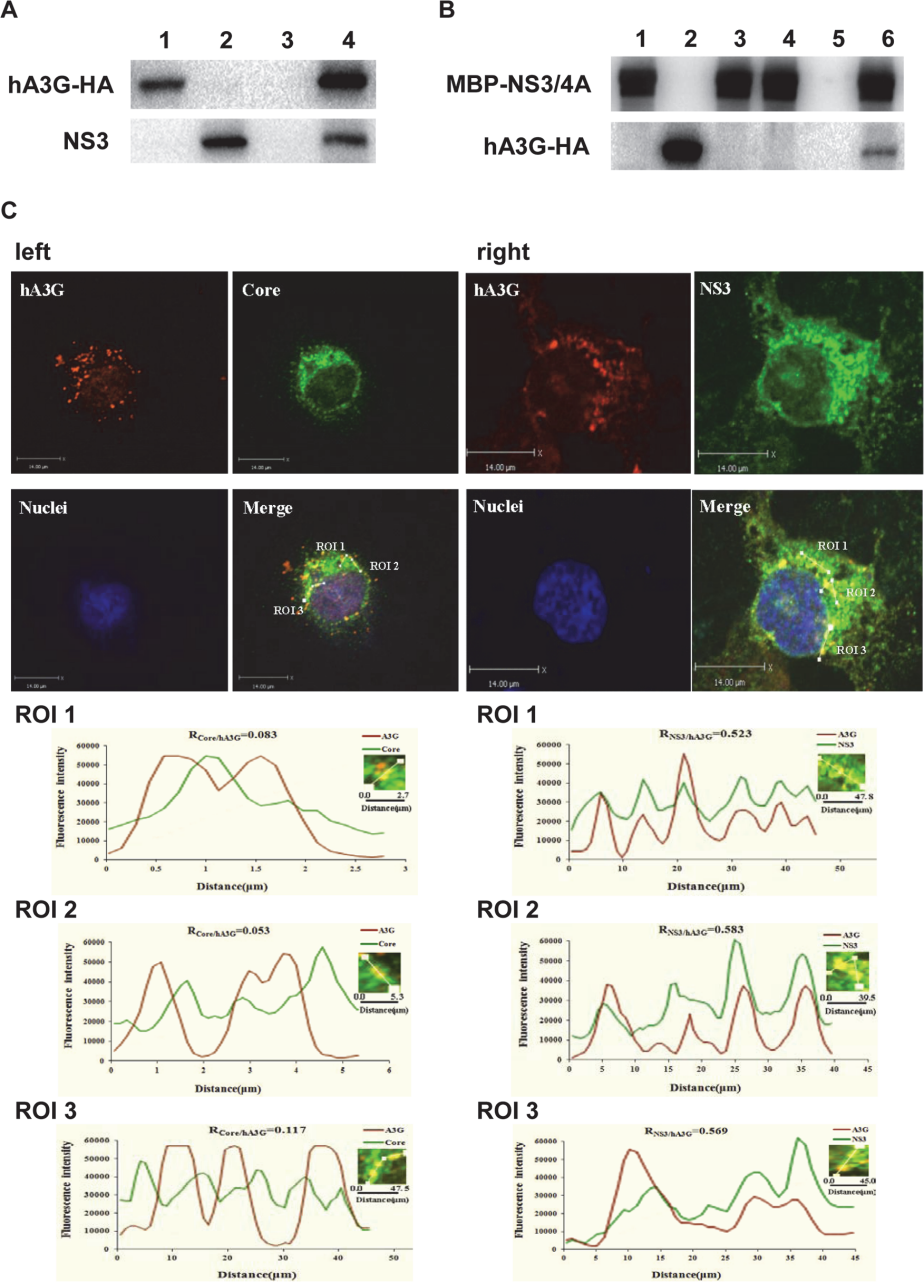
Protein-protein interaction between hA3G and HCV NS3. (A) NS3 protein in immunoprecipitates was detected with anti-HA antibody (hA3G-HA) by western blot. Supernatants from cell lysates of HCV-infected Huh7.5 cells transfected with phA3G-HA expression vector (lane 4) or plasmid control (lane 3) were incubated, in turn, with HA-specific antibody and Protein A Agarose; the agarose was washed with DPBS, then the immunoprecipitates were boiled and analyzed with western blot using anti-HA or anti-NS3 as a probe. Lane 1, hA3G-HA positive control; lane 2, NS3 positive control. (B) hA3G was bound to maltose-binding protein (MBP)-NS3/4A *in vitro*. MBP-NS3/4A was firstly bound to amylose resin column, followed by interaction with cell lysates of the Huh7.5 cells transfected with hA3G-HA expression vector or plasmid control; after eluted with eluting buffer, the elutriants were analyzed with western blot probed with anti-NS3 or anti-HA antibody. Lane 1, MBP-NS3/4A positive control; lane 2, hA3G-HA positive control; lane 3, negative control (elutes of lysates of Huh7.5 cells transfected with control plasmid); lane 4, negative control (elutes of free-cells lysates); lane 5, negative control (elutes of lysates of Huh7.5 cells transfected with plasmid hA3G-HA,using amylose resin column with no pre-binding of MBP-NS3/4A); lane 6, sample (elutes of lysates of Huh7.5 cells transfected with plasmid hA3G-HA). (C) Intracellular distribution of hA3G (red signal), HCV core (green signal, left) as well as HCV NS3 (green signal, right) in HCV-infected Huh7.5 cells was analyzed with immune fluorescent staining; blue signal: nucleus. The histograms show the fluorescence intensity analysis results from ROI 1,ROI 2 and ROI 3 in the merged panel using Volocity Demo software. Correlation coefficients for each couple of intensity values (R_Core/hA3G_ or R_NS3/hA3G_) are shown. The average correlation coefficient is 0.084 ± 0.032 for hA3G and HCV core (R_Core/hA3G_) and 0.558 ± 0.031 for hA3G and NS3 (R_NS3/hA3G_).

Our early evidence came from an immunoprecipitation experiment. HCV-infected Huh7.5 cells were transfected with the hA3G-HA vector, followed by 48hrs incubation. Then, the cell lysates were incubated in turn with HA-specific antibody and Protein A Agarose, followed by washing. SDS-PAGE electrophoresis was done to separate proteins within the immunoprecipitates. Protein bands in the gel were extracted and purified for protein sequencing. The interesting finding was that the HCV NS3 specific peptide fragment sequences KVPVAYAAQGYKV, KCGAVDLYLVTRN and KSIDFIPVETLDVVTRS were detected in the bands, suggesting that HCV NS3 was bound to hA3G-HA. Western blot showed that the immunoprecipitates indeed contained HCV NS3 protein (Fig. 1A), consistent with the protein sequencing results.

Next, in a reversed fashion, we first used maltose-binding protein (MBP)-NS3/4A to bind to an amylose resin column, then the cell lysates of Huh7.5 cells transfected with hA3G-HA expressing vector were added to the column. Again, MBP-NS3/4A as well as hA3G-HA were both detectable in the eluted fraction by western blot (Fig. 1B), confirming the interaction between the two proteins.

We then examined the intracellular distribution of the two proteins using laser scanning confocal techniques. Region of interest (ROI) analysis for 3 different areas in the cytoplasm (ROI_1,2,3_) was done in order to view the possible interaction between the study proteins. The analysis was done with Volocity Demo software. As shown in Fig. 1C, while the HCV core protein (green signal) localized differently from hA3G (red signal) [the average correlation coefficient between core and hA3G (R_Core/hA3G_) was 0.084 ± 0.032; Fig. 1C, left], HCV NS3 (green) showed its signal peak at spots almost overlapping with those of hA3G (red) [the average correlation coefficient between NS3 and hA3G (R_NS3/hA3G_) was 0.558 ± 0.031; Fig. 1C, right]. This experiment provided strong support for the results obtained from the immunoprecipitation experiments.

### hA3G protein bound directly to HCV NS3

**Fig 2:**
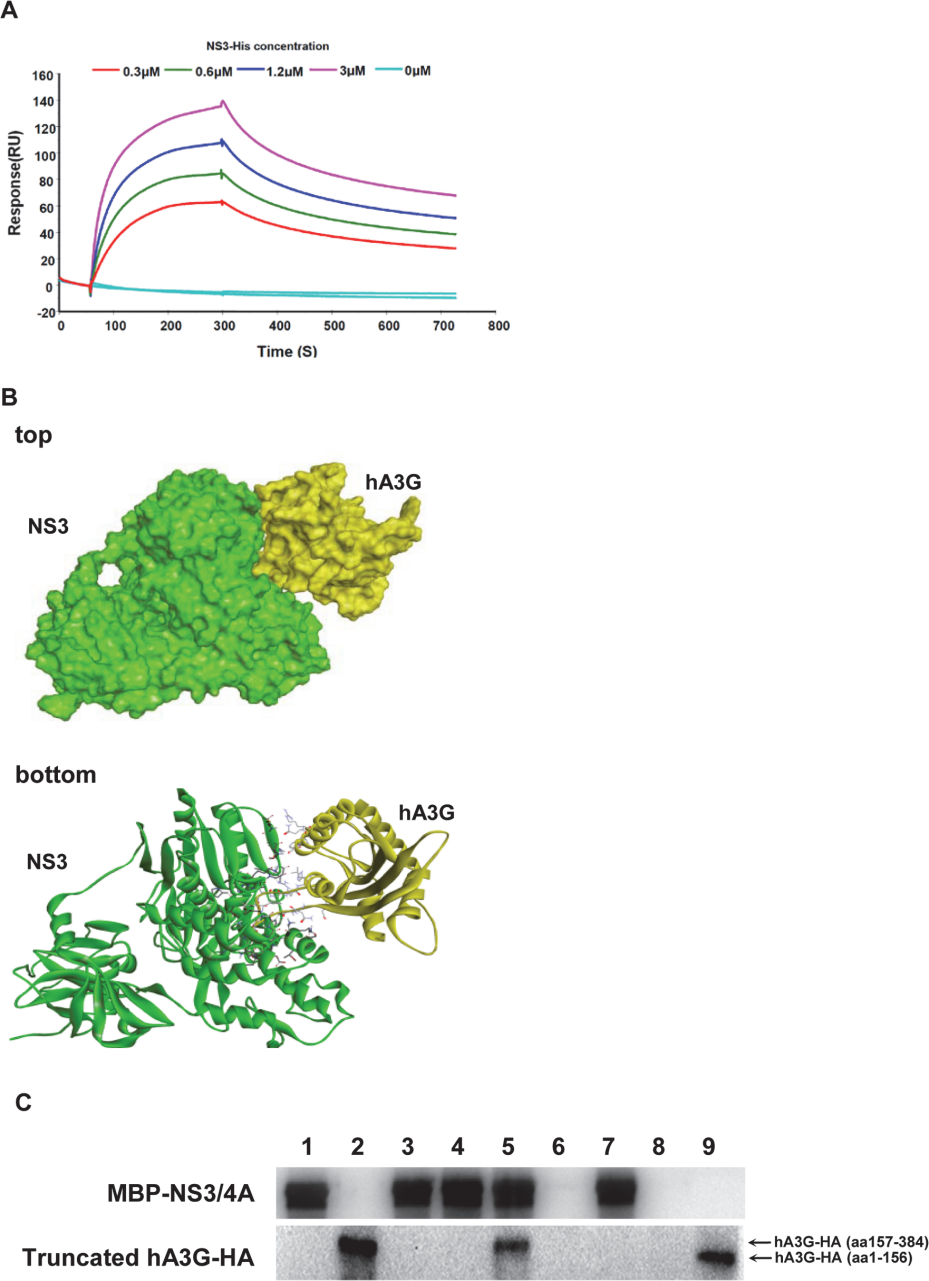
hA3G protein directly bound HCV NS3. (A) The binding of NS3-His (different concentrations or control) to hA3G-HA were measured with the surface plasmon resonance using BIAcore T100 biosensor system. (B) Computer-simulated docking complex structure between hA3G and NS3 (Pose 11, which was selected from over 2000 poses) were displayed in surface (top) and solid ribbon forms (bottom). (C) C-terminal of hA3G bound MBP-NS3/4A, but N-terminal did not. The MBP-NS3/4A was firstly bound to amylose resin column, followed by interaction with cell lysates of the 293T/17 cells, which were transfected with vectors expressing truncated fraction of hA3G-HA; the western blot analysis for elutes was done with anti-NS3 or anti-HA antibody. Lane 1, MBP-NS3/4A protein control; lane 2, hA3G-HA (aa157-384) positive control; lane 3, negative control (elutes of lysates of 293T/17 cells transfected with control plasmid); lane 4, negative control (elutes of free-cells lysates); lane 5, sample (elutes of lysates of 293T/17 cells transfected with plasmid hA3G-HA (aa157-384)); lane 6, negative control (elutes of lysates of 293T/17 cells transfected with plasmid hA3G-HA (aa157-384), using the amylose resin column with no pre-binding of MBP-NS3/4A); lane 7, sample (elutes of lysates of 293T/17 cells transfected with plasmid hA3G-HA (aa1-156)); lane 8, negative control (lysates of 293T/17cells transfected with plasmid hA3G-HA (aa1-156), using the amylose resin column with no pre-binding of MBP-NS3/4A); lane 9, hA3G-HA (aa1-156) positive control.

Another question was whether or not the binding between hA3G and NS3 is a direct interaction. Thus, purified hA3G-HA protein was adhered on chips and reacted with different concentration of purified NS3-His in a mobile phase using a BIAcore system. As shown in Fig. 2A, a direct binding between hA3G and NS3 was found and in a dose-dependent manner, suggesting a straight interaction between the two proteins. Although RNA seems to be an important component in the interaction between A3G and HIV Gag [20], participation of RNA might not be essential in the interaction between hA3G and NS3.

Binding mode between hA3G and NS3 was analyzed as well using Software Discovery Studio 3.0. Docking analysis between hA3G (amino 190–380, PDB code: 3IQS) and the homology model of NS3 (amino 1–631, PDB code: 3O8B) was done. On the basis of the hydrophobic nature of the studied proteins as well as comprehensive analysis, Pose 11 was selected from over 2000 poses and considered to be the most likely one to mimic the interaction between hA3G and NS3 (Fig. 2B, top). Computer simulation predicted that fragments of amino acids Gln245-Ala246, His248-His257, Leu260, Phe289-Ser290, Gln293 and Arg320 in hA3G, as well as Pro230, Val232, Thr254-Met260, Thr269-Lys272, Ala275, Thr298, Glu493, Asp496-Ala497, Ala500-Tyr502 and Ser548-Ala558 in NS3 are likely the binding sites according to the contact surface area (Fig. 2B, bottom). It appeared that the hA3G C-terminus was the domain which binds to a fragment roughly two-thirds the length of the protein from the C-terminal of NS3, according to computer calculation.

To confirm the docking results, expression vectors of the truncated hA3G-HA fraction (C-terminus or N-terminus) were respectively transfected into the 293T/17 cells, which is null of hA3G [3]; then the cell lysate was applied onto the amylose resin column which was pre-bound with MBP-NS3/4A protein. NS3 in the amylose resin column directly captured the hA3G C-terminus peptide (amino acid 157–384), whereas the hA3G N-terminus peptide (amino acid 1–156) was not detectable in the immunoprecipitates (Fig. 2C). The result indicated that the hA3G C-terminus was the domain that bound HCV NS3 protein, agreeing with the Pose 11 interaction model. To further validate the binding sites, experiments using mutated NS3 at positions described in Fig. 2B are needed.

### HCV NS3 helicase activity was inhibited by hA3G

**Fig 3:**
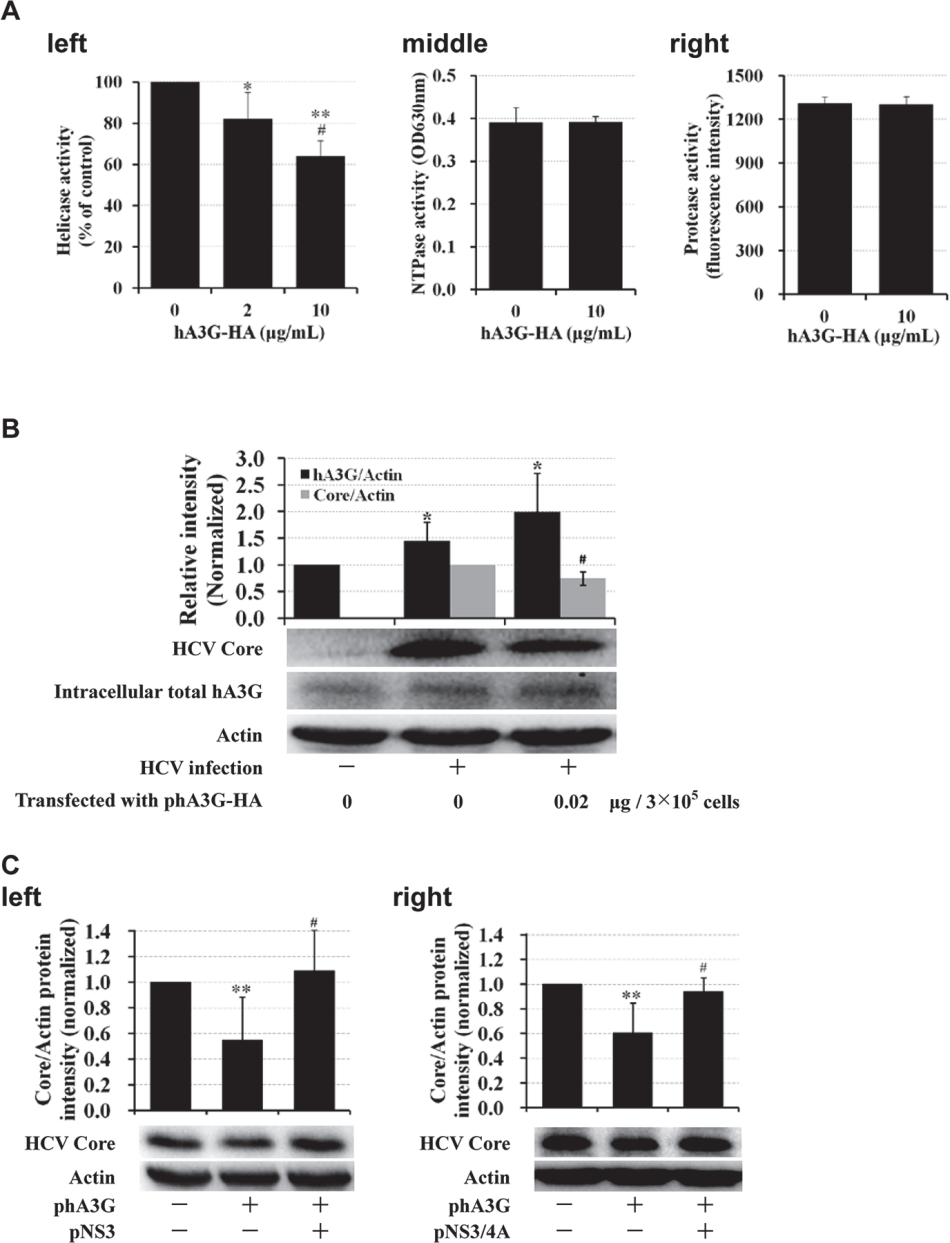
Inhibitory effect of hA3G on enzymatic activities of HCV NS3. (A) hA3G inhibited activity of HCV NS3 helicase (left) in a cell-free reaction system, however, it showed no significant impact on NTPase (middle) and serine protease (right) activities of NS3, even at concentration of 10μg/mL. Presented were mean ± SD (n = 3). *P<0.05 and **P<0.01: treated *vs*. 0μg/mL hA3G-HA group; ^#^ *P*<0.05: 10μg/mL *vs*. 2μg/mL hA3G-HA group. (B) Ectogenous hA3G restricted HCV replication in Huh7.5 cells. HCV infected (with 45 IU/cell) or uninfected Huh7.5 cells were transfected with phA3G-HA or plasmid control; 48hrs later, the cell lysates were extracted and analyzed with western blot to detect HCV core (top band), intracellular total hA3G (middle band) and internal-control Actin (bottom band). The scanned intensity histogram showed the average value of western blot (n = 3). The bands presented below showed the results of a representative experiment. *P<0.05: HCV infection with or without phA3G-HA transfection *vs*. 0μg/mL phA3G-HA with no HCV infection; ^#^ *P*<0.05: HCV infection with 0.02μg/mL phA3G-HA transfection *vs*. HCV infection alone. (C) Ectogenous NS3 reversed the restrictive activity of hA3G on HCV. HCV infected (with 45 IU/cell) Huh7.5 cells were co-transfected with 0.2μg phA3G and 0.2μg pNS3 (left)(or pNS3/4A (right)); 72hrs later, the cell lysates were extracted and analyzed with western blot. The histogram showed the average density value of western blot (n = 2). The bands presented below showed the results of a representative experiment. **P<0.01: transfected with phA3G *vs*. untransfected with phA3G and pNS3 (or pNS3/4A); ^#^ *P*<0.05: co-transfected with phA3G and pNS3 (or pNS3/4A) *vs*. transfected with phA3G alone.

HCV NS3 protein is a multifunction viral enzyme, with serine protease activity at the one-thirds of the N-terminus, and NTPase as well as helicase activities at the two-thirds of the C-terminus. These viral enzymes play a key role in HCV replication. To understand the NS3 enzymatic activity changes that occur after binding with hA3G, NS3 activity (controlling serine protease, helicase and NTPase) was measured with a fluorescent resonance energy transfer assay, fluorescent helicase assay and malachite green phosphate assay, respectively, in the absence or presence of purified hA3G-HA. Treatment of NS3 with purified hA3G-HA reduced NS3 helicase activity. The inhibitory effect was dose-dependent, with 36.1% inhibition of helicase activity at 10μg/mL(*P*<0.01) and 17.8% at 2μg/mL(*P*<0.05), respectively (Fig. 3A, left). Although the inhibition on NS3 helicase by hA3G was moderate in the cell-free system, transfection of the HCV infected cells with hA3G expression vector increased intracellular hA3G level and significantly inhibited HCV replication (Fig. 3B; *P*<0.05). In contrast, the serine protease activity was not obviously altered by hA3G at concentration of 10μg/mL (about 0.23μM) (Fig. 3A, right), probably because it associates with the N-terminal function of NS3. The result agreed with the analysis from computer simulations, and it appeared that binding of hA3G at the C-terminus of NS3 had almost no impact on the N-terminal stereochemical structure of NS3. NTPase activity remained unchanged in the presence of hA3G (Fig. 3A, middle) and the reason is unclear. We speculate that it might be due to an ineffective interaction between hA3G and the domain responsible for NTPase activity, or that the configuration change on NS3 by hA3G might not interrupt NTPase’s activity. To learn whether addition of NS3 could rescue HCV replication from hA3G attack, we co-transfected the HCV infected cell with hA3G and NS3 (or NS3/4A) expression vectors. The results show that transfection with hA3G plasmid reduced HCV replication (*P*<0.01), while co-transfected with NS3 plasmid, HCV replication recovered (Fig. 3C, left; *P*<0.05). Co-transfected with NS/4A plasmid generated a similar result (Fig. 3C, right; *P*<0.05).

### Host intracellular hA3G was transiently increased in the early stage of HCV infection

**Fig 4:**
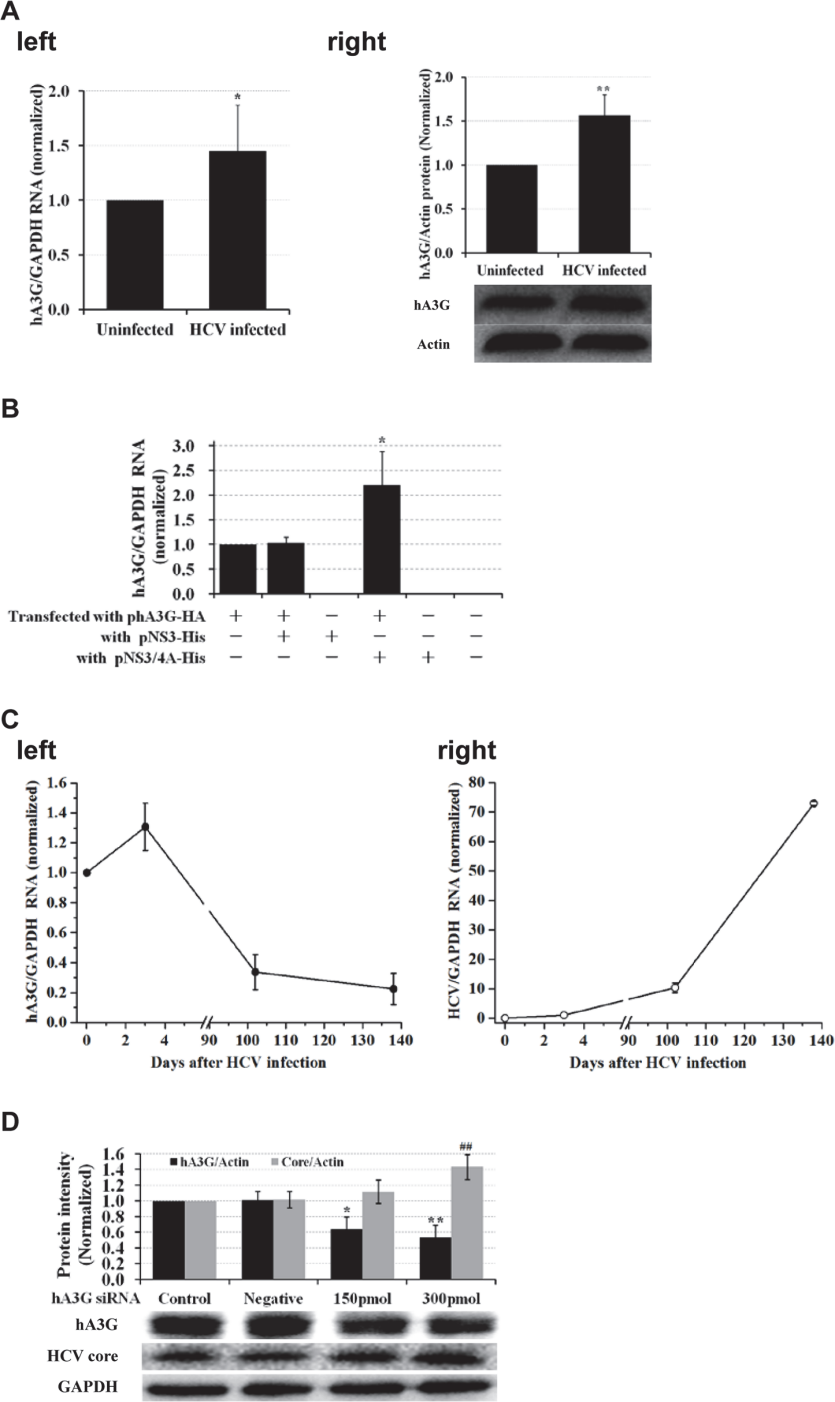
HCV infection caused a transient increase of intracellular hA3G. (A) Intracellular hA3G expression was increased at either RNA (left, n = 3) or protein (right, n = 3) level in Huh7.5 cells 3 day after HCV infection. The bands presented below showed the results of a representative experiment. *P<0.05 and **P<0.01: HCV infected cells *vs*. uninfected. (B) Intracellular hA3G was increased by NS3/4A. Real time qRT-PCR was used to analyze hA3G mRNA in the 293T/17 cells, after 48 hrs co-transfection with phA3G-HA and pNS3-His or pNS3/4A-His. Shown was the average value (n = 3). *P<0.05: co-transfected with phA3G-HA and pNS3/4A-His *vs*. transfected with phA3G-HA alone. (C) Long-term culture of HCV-infected Huh7.5 cells caused a decline of the intracellular hA3G and increase of HCV in cells (left, hA3G RNA; right, HCV RNA). (D) Specific siRNA for hA3G enhanced HCV replication in HCV infected Huh7.5 cells. Density histogram showed the average values of 3 independent experiments. The bands presented below showed the results of a representative experiment. A non-relevant siRNA at concentration of 150 pmol was used as a negative control in this experiment. *P<0.05, **P<0.01 and ^##^ *P*<0.01: hA3G silenced *vs*. control group.

HCV infection caused an increased expression of intracellular hA3G at either mRNA (Fig. 4A, left; *P*<0.05) or protein level (Fig. 4A, right; *P*<0.01), consistent with the observation in HCV infected patients [21] as well as HCV positive patients co-infected with hepatitis B virus (HBV) [22]. It might represent an activation of host innate defensive system after HCV attack, as hA3G is an innate antagonist of HCV [7]. The mechanism behind the increase of hA3G in HCV infected cells seems to be associated with NS5A [21]. In the present study, we also found that transfection of NS3 did not increase hA3G levels; however, transfection of NS3/4A enhanced hA3G expression (Fig. 4B; *P*<0.01). In HCV-infected Huh7.5 cells, intracellular hA3G RNA (Fig. 4C, left) increased after HCV infection and then decreased with time in a long-term culture; accordingly, HCV RNA level (Fig. 4C, right) significantly increased after over 100 days cultivation of the infected cells, in which hA3G level largely decreased. It suggests an unknown mechanism of the virus that protects HCV from the inactivation effect of hA3G. It could be one of the factors responsible for disease progress in HCV infection in clinic. In fact, knockdown of hA3G expression with siRNA enhanced HCV replication in a dose-dependent manner (Fig. 4D; *P*<0.05), providing further evidence in support of the results in the long-term culture experiment (Fig. 4C).

## Discussion

HCV is a positive single-stranded RNA virus belonging to the *Flaviviridae* family and is a causative agent for hepatitis C around world. Interferon-alpha in combination with ribavirin was the standard treatment for chronic hepatitis C in the past few decades. In 2011, two HCV serine protease inhibitors telaprevir and boceprevir were approved by the US FDA for anti-HCV treatment. Recently, important progress have been made, including the polymorphism in IL-28B gene [23], which relates to HCV sensitivity to interferon treatment and provides a molecular indication for personalized therapy in anti-HCV therapy. More importantly, in 2013 two new drugs for HCV have been approved by the US FDA: one is serine protease inhibitor simeprevir, and the other is RNA-dependent RNA polymerase inhibitor sofosbuvir. Even though, the overall therapeutic effect in HCV-infected patients is sub-optimal [24], and HCV drug-resistant mutation under therapeutic pressure might be a significant challenge for the drugs in the future [25], as these new drugs all specifically target HCV replicative enzyme protease or polymerase.

At least three approaches have been investigated to overcome viral drug-resistant mutation. The strategies include combination therapy with drugs targeting different viral molecules [26], identification of conservative viral sequences as targets for drug screening [27], and discovery of host-based antiviral mechanisms [28]. In the past few years, the interaction between HCV and host molecules (proteins or RNAs) have been actively investigated, and some of them are of interest in overcoming HCV drug-resistance [29]. Recently, as the host protein hA3G has become an attractive machinery to control viral replication, enhancing hA3G (by hA3G stabilizer or by interruption of the interaction between hA3G and HIV-1 vif) has been considered as a new strategy to discover anti-viral agents [7–9, 30–31]. Indeed, our previous results showed that hA3G stabilizers were strongly active against HCV [7], thus clarification of anti-HCV molecular mechanism of hA3G might help us to find new anti-HCV candidates working through hA3G.

Host hA3G inhibits HIV-1 mainly through catalyzing cytidine deamination of viral DNA on the minus strand which results in G-to-A hypermutations in reverse transcripts [4, 5]. However, deamination-independent antiviral effects of hA3G were also documented in infections of HIV-1, HBV, HCV, as well as others viruses [7, 12] including negative-strand RNA viruses such as measles, mumps and respiratory syncytial virus [12]. The mode of action of hA3G remains unclear. This study investigated the anti-HCV mechanism of hA3G, and showed that it appeared to inhibit HCV NS3 helicase activity through a direct binding of its C-terminus to the C-terminus of NS3.

In summary, HCV infection enhances cell production of hA3G, which then decreases the activity of HCV NS3 through a mechanism described above; on the other hand, to overcome hA3G’s restrictive effect, HCV gradually develops an unidentified mechanism that reduces intracellular hA3G level and secures HCV replication. This viral-host interaction process could be considered a new target to discover anti-HCV drugs working through host mechanism. We believe that understanding the antiviral mechanism of hA3G on HCV might provide an interesting approach to explore hA3G stabilizers as a new class of anti-HCV drugs in the future.
